# Discordance of Dual-Tracer PET/CT with Histopathology in a Grade I Pancreatic Neuroendocrine Tumor: A Diagnostic Conundrum

**DOI:** 10.1055/s-0045-1812058

**Published:** 2025-10-04

**Authors:** Sarina Shah, Keerti Sitani, Gauri Deshpande, Sandip Basu

**Affiliations:** 1 Radiation Medicine Centre, Bhabha Atomic Research Centre, Tata Memorial Hospital Annexe, Parel, Mumbai, Maharashtra, India; 2 Homi Bhabha National Institute, Mumbai, Maharashtra, India; 3 Department of Pathology, Tata Memorial Centre, Parel, Mumbai, Maharashtra, India

**Keywords:** neuroendocrine tumors, ^177^ Lu-DOTATATE PRRT, ^68^ Ga-DOTATATE PET/CT, somatostatin receptor (SSTR), fluorine-18-deoxyglucose ( ^18^ FDG), dual-tracer PET/CT

## Abstract

**Objectives:**

Neuro endocrine tumors (NETs) demonstrate complex biology where imaging and histology can at times show discordance in a real-world scenario.

**Materials and Methods:**

We herein present a patient of grade I pancreatic NET showing atypical low somatostatin receptor expression on ^68^ Ga-DOTATATE PET/CT (positron emission tomography/computed tomography) but intense fluorine-18-deoxyglucose ( ^18^ FDG) avidity, and when interpreted together, these findings showed discordance with histopathology.

**Results:**

The case highlights the crucial role of dual-tracer PET/CT in navigating the tumor biology and landscape of NET and the need for novel strategies to complement traditional histopathology that would enable better therapeutic decision-making.

## Introduction

Neuroendocrine neoplasms (NENs) are a rare heterogeneous group of tumors originating from secretory neuroendocrine cells, which are distributed throughout the body and as a result, NENs can arise in virtually any organ. The past few decades have seen an increasing incidence of neuroendocrine tumors (NETs), owing largely to greater clinical awareness, improved diagnostic techniques, and a deeper understanding of their biology. Unlike many common malignancies, where prognosis primarily depends on tumor histology and stage, NENs exhibit highly variable biological behavior and outcomes, depending not only on stage but also on factors such as tumor differentiation, grade, primary site, receptor expression, and functional status. 1

As per the recent World Health Organization (WHO) Classification (2022), NENs are broadly categorized into well-differentiated NET and neuroendocrine carcinoma (NEC). NETs are further stratified into grades I–III based on the Ki-67 proliferative index, with prognosis declining as the tumor grade increases. A hallmark feature of well-differentiated NETs is somatostatin receptor (SSTR) expression, which serves as both a diagnostic marker and a therapeutic target. SSTR imaging enables tumor staging, treatment response assessment, and guides therapy selection—including cold somatostatin analogues and peptide receptor radionuclide therapy (PRRT). As NETs de-differentiate, they frequently show a decrease in SSTR expression, a phenomenon clearly demonstrable on SSTR-directed PET imaging. 2

Dual-tracer positron emission tomography/computed tomography (PET/CT) has emerged as a very useful tool for comprehensive in vivo tumor characterization. While single-site biopsies often inadequately capture the complex intratumoral and intertumoral heterogeneity, molecular PET imaging may provide a holistic whole-body tumor assessment. Currently, SSTR-targeted PET/CT utilizes Gallium-68 labelled conjugated peptides, primarily Tyr3-octreotate ( ^68^ Ga-DOTATATE), or its variants like DOTATOC and DOTANOC. Complementary to this, fluorine-18-deoxyglucose ( ^18^ FDG)-PET/CT evaluates tumor metabolic activity, offering additional prognostic information.

The prognostic value of dual-tracer PET/CT in NETs has been demonstrated in prior studies. Typically, tumors exhibiting high SSTR expression with absent ^18^ FDG uptake across all metastatic sites portend an indolent course and favorable prognosis. Conversely, lesions with low SSTR expression and intense ^18^ FDG uptake suggest aggressive tumor biology with heightened metabolic activity and consequently poorer outcomes. 3 4

We present a case of grade I well-differentiated pancreatic NET demonstrating discordant functional imaging features: atypically low SSTR expression with high ^18^ FDG uptake at initial staging, which subsequently progressed to grade II NET.

## Case History

A 43-year-old woman with no significant comorbidities presented with a 1-year history of intermittent mid-abdominal pain managed occasionally with analgesics. Contrast-enhanced abdominal CT revealed a large mass (4.2 × 4.6 × 11.2 cm) involving the pancreatic head and uncinate process, with associated conglomerated lymphadenopathy. The lesion demonstrated locally aggressive features, including compression of the common bile duct and main pancreatic duct, abutment of superior mesenteric vein, and infiltration of the second part of duodenum. Laboratory evaluation showed normal liver function tests and normal levels of serum carcinoembryonic antigen and carbohydrate antigen 19-9. She underwent endoscopic retrograde cholangiopancreatography and biliary stenting during which a biopsy sample was obtained. The histopathological analysis revealed a well-differentiated grade I NET with Ki-67 index of 2%. Her serum chromogranin A level was 122 ng/mL (60–100 ng/mL).

**Fig. 1:**
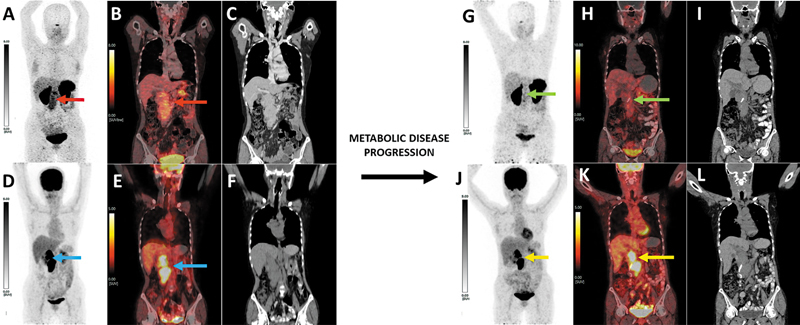
Baseline ^68^ Ga-DOTATATE PET/CT maximum intensity projection (MIP) image ( **A** ) and fused coronal PET/CT ( **B** ) with corresponding CECT slice in soft tissue window ( **C** ) showing the large infiltrative pancreatic lesion and conglomerated lymphadenopathy (red arrows) with SUVmax: 8.12 (Krenning score 3). Concurrent ^18^ FDG PET/CT MIP ( **D** ) and fused coronal PET/CT ( **E** ) and coronal CT slice ( **F** ) showing intense ^18^ FDG concentration in the same lesion, SUVmax: 7.30 (blue arrows). The baseline NETPET score is P4. At 4 months from the completion of the 2nd cycle of 177Lu-PRRT, repeat dual PET/CT was obtained for assessing response. Response ^68^ Ga-DOTATATE PET/CT ( **G** : MIP, **H** : fused coronal PET/CT, **I** : coronal CT) and ^18^ FDG PET/CT ( **J** : MIP, **K** : fused coronal PET/CT, **L** : coronal CT) demonstrate almost absent SSTR expression in the lesion (green arrows), SUVmax: 5.52 (Krenning score 1), with intense ^18^ FDG concentration (yellow arrows), SUVmax: 11.74. NETPET score at response assessment is P5. CECT, contrast-enhanced computed tomography; NET, metastatic neuroendocrine tumor; PET/CT, positron emission tomography/computed tomography; SSTR, somatostatin receptor.

**Fig. 2:**
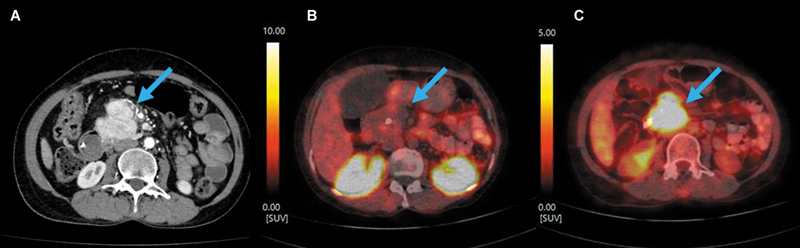
Axial slices of CECT ( **A** ), fused ^68^ Ga-DOTATATE PET/CT ( **B** ), and fused ^18^ FDG PET/CT ( **C** ) at the time of response assessment depicting the enhancing lobulated pancreatic head lesion with absent SSTR expression and intense ^18^ FDG concentration. CECT, contrast-enhanced computed tomography; ^18^ -FDG, fluorine-18-deoxyglucose; PET/CT, positron emission tomography/computed tomography; SSTR, somatostatin receptor.

**Fig. 3:**
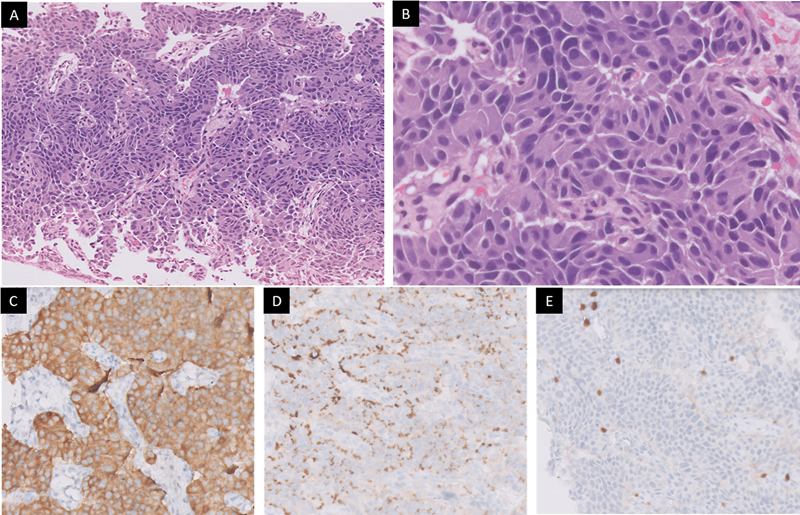
( **A** ) Biopsy cores showing tumor arranged in organoid pattern (H&E, 100 × ). ( **B** ) The tumor cells show relatively monomorphic nuclei with focal sudden anisonucleosis and inconspicuous mitotic activity (H&E, 400 × ). ( **C** ) Tumor cells are diffusely positive for synaptophysin (DAB, 200 × ) and ( **D** ) chromogranin (DAB, 200 × ). ( **E** ) Mib1 labeling index is 3 to 5% in the highest proliferating areas.

Baseline staging ^68^ Ga-DOTATATE PET/CT showed an SSTR-expressing primary lesion in the pancreas, with tracer uptake slightly more than the liver. Given this relatively lower SSTR avidity for a grade 1 NET, ^18^ FDG PET/CT was also done at baseline, which showed high-grade ^18^ FDG concentration in the above mass. As surgical resection was not feasible, she was counselled regarding the therapeutic options of oral chemotherapy with capecitabine and temozolomide (CAPTEM), along with a trial of PRRT in view of Krenning 3 uptake. She received two cycles of Lutetium-177 DOTATATE PRRT ( ^177^ Lu-DOTATATE PRRT). At 4 months from the last cycle of PRRT, she was clinically asymptomatic with a serum chromogranin A level of 64.4 ng/mL, and on her response evaluation, dual-tracer PET/CT demonstrated a significant reduction in SSTR expression, the uptake now being lower than the liver (Krenning 1). ^18^ FDG-PET/CT showed approximately 60% increase in metabolic activity as compared to the baseline ( Figs. 1 and 2 ). As there was a suspicion of progression to a higher grade NET or NEC, a repeat biopsy of the primary lesion was obtained. The repeat histopathological analysis showed a well-differentiated grade 2 NET, with Ki-67 index of 3 to 5%. A second review of the biopsy specimen was in agreement with the above findings ( Fig. 3 ). Further ^177^ Lu-DOTATATE PRRT was deferred and she was referred to the medical oncologist for chemotherapy. She received seven cycles of CAPTEM chemotherapy, following which her disease status was stable at 1 year.

## Discussion

NETs constitute a rare malignancy subtype originating from specialized neuroendocrine cells distributed throughout the body. These neoplasms exhibit hybrid features of both neural and endocrine differentiation. 5 Approximately 70 to 75% arise in the gastroenteropancreatic (GEP) system, followed by pulmonary sites, with 20 to 35% being functionally active, leading to characteristic clinical syndromes. 6 Traditionally, tumor differentiation and tumor grade have been recognized as important determinants of the clinical nature and prognosis of NETs.

Differentiation indicates the extent to which tumor cells resemble the cells of origin, and the tumor grade, as measured by the mitotic rate/the Ki-67 index, represents the proliferative activity of the tumor cells. The WHO has broadly divided epithelial NETs into well-differentiated NETs and NECs based on their morphology. NETs were further divided into grade I (Ki-67 index < 3%), grade II (Ki-67 index 3–20%), and grade III (Ki-67 index > 20%). NET cells typically show strong immunohistochemical positivity for chromogranin A and synaptophysin, along with high hormone and SSTR expression, whereas NECs show low-grade or absent hormone and SSTR expression. 2

The primary and metastatic sites of NET are known to exhibit a heterogenous nature. Variations in chromogranin, synaptophysin, and Ki-67 expression between the primary lesion and metastatic sites have been documented, with maximal variation of Ki-67 shown by metachronous metastatic sites. Furthermore, it has been documented that over the course of disease progression and treatment, NETs can show a change in their behavior, as depicted by a change in their Ki-67 index, obtained via a repeat biopsy, predominantly leading to disease upstaging. 7 8

Tran et al demonstrated through RNA sequencing that in pancreatic NET, hepatic metastasis had a 1.9-fold lower SSTR expression than their primary tumors; however, they did not find a significant difference in SSTR expression between the primary and nodal metastases. They found that the decreased SSTR expression led to increased cell growth and proliferation via the activation of Akt/mTOR pathway, which is known to play a major role in tumorigenesis of many cancers. 9

For years, only SSTR imaging has been used for the diagnosis and staging of well-differentiated NETs; however, as the concept of tumor heterogeneity has gained popularity, the role of FDG PET/CT in the prognostication of NETs has become increasingly recognized.

Chan et al devised a 5-point scale NETPET score for stratification of GEP-NETs based on dual PET/CT for disease prognostication, which was further validated in a multicentric study. They found that overall survival (OS) and time to progression (TTP) correlated well with the NETPET score and histological tumor grade. A higher NETPET score (score P5: SSTR negative/FDG positive) was associated with a worse prognosis than that of P1 (SSTR positive/FDG negative). The study showed a median OS/TTP of 101.8/25.5 months for P1, 46.5/16.7 months for P2–4, and 11.5/6.6 months for P5. 10 11 This scoring system also helps in selecting candidates who would benefit from PRRT as demonstrated by Zhang et al from a retrospective analysis of 495 patients who were stratified as per the NETPET score and then received PRRT. The study enforced the evidence that FDG-negative status was an independent prognostic factor for both OS (0.5-fold decrease in death) and progression-free survival (0.7-fold decrease in the risk of progression). 4

Also, since the Ki-67 index plays a crucial role in determining the chemotherapy regimen and it can be an independent prognostic marker, a re-biopsy of the lesion is often essential. A published study in 2016 examined the correlation between Ki-67 indices from initial tissue samples and subsequent resection specimens in 36 patients. While there was a strong overall correlation ( *r* = 0.95, *p* < 0.001), discrepancies were noted in approximately 39% of cases. Notably, 27.8% of initial biopsies underestimated the tumor grade compared to the resected specimens. This underestimation could lead to less aggressive treatment approaches than necessary. The study concluded that a low Ki-67 index from an initial biopsy should be interpreted cautiously, especially if clinical, biochemical, or radiological evidence suggests a more aggressive disease pathology. 12

While current biomarkers (e.g., chromogranin A, Ki-67 index) and imaging methods remain essential for NET management, they exhibit significant limitations in prognostic stratification, therapeutic monitoring, and predicting response to PRRT. A recent prospective study (the LuMEn trial) conducted by Mileva et al demonstrated that tumor-absorbed dose plays a fundamental prognostic role. The researchers performed three-point dosimetry using ^177^ Lu-DOTATATE SPECT/CT after each cycle of PRRT in patients with advanced GEP-NETs. Patients who achieved at least 35 Gy tumor-absorbed dose in all target lesions after the first cycle of ^177^ Lu-PRRT had significantly longer progression-free survival than those in whom even one target lesion received less than 35 Gy. This simplified dosimetry approach is clinically practical and may help identify patients who would benefit from a higher therapeutic dose early in treatment, potentially improving outcomes with PRRT and identifying those who may benefit from other therapeutic modalities. 13

Keeping this in mind, even in our case, as the tumor characteristics changed over the course of treatment, as evidenced by the unexpectedly low SSTR expression, we sought a repeat biopsy. Although the tumor was upstaged from grade I to grade II, as shown by an increase in Ki-67 from 2 to 5%, the lack of SSTR uptake on dual PET/CT was highly unusual. Histopathology and dual-tracer PET/CT findings are usually concordant, and histopathological grading is a reliable prognostic marker. However, in real-world scenarios, outliers do exist, such as in this case, low SSTR expression and high FDG concentration in grade I NET. Conversely, high SSTR expression and low FDG concentration in grade III NET have also been documented. In such discordant cases, histopathology may not reflect the tumor biology as reliably as dual-tracer PET/CT, which can assess the whole-body tumor burden in vivo. Biopsy and tissue-based analyses are limited by sampling size and localized tumor site grading. 14 In addition, because it is an invasive procedure, it cannot be performed repeatedly.

Emerging liquid biopsy technologies include revolutionizing precision oncology through detection of circulating biomarkers such as circulating tumor cells, circulating free/tumor DNA, and transcriptome analysis. Although still in developmental stages, preliminary studies demonstrate that such approaches can provide deeper insight into the spatiotemporal dynamics of cancer progression and aid in personalized therapy selection through molecular profiling, along with a noninvasive assessment of treatment response. 15 16 Similarly, texture analysis using advanced techniques to quantify tissue heterogeneity based on pixel intensity distributions offers a promising approach in evaluating challenging or discordant cases. 17 Radiomics, by extracting and analyzing these imaging features, holds significant potential as a valuable tool in tumor characterization, aiding in prognostic assessment and evaluation of treatment response. 18

## Conclusion

In summary, the present case of a well-differentiated pancreatic NET with discordant SSTR/FDG-PET findings, demonstrating aggressive metabolic features despite maintained histological differentiation, is not uncommon in clinical practice. It highlights the evolving role of dual-tracer PET/CT in assessing tumor biology beyond the histological classification, as well as critical gaps in current biomarker paradigms and the need for further advanced diagnostic biomarkers in addition to the traditional Ki-67 index.
